# Geospatial Analysis of Rurality and Food Banks in Appalachian Ohio

**DOI:** 10.13023/jah.0303.09

**Published:** 2021-07-25

**Authors:** Cooper T. Johnson, Rebecca Fischbein, Kristin R. Baughman

**Affiliations:** Northeast Ohio Medical University

**Keywords:** Appalachia, food insecurity, food pantry, mapping, rural

## Abstract

**Introduction:**

Food insecurity is a problem for individuals across Ohio, including those living in Appalachia. Adequate access to resources that help combat food insecurity is important for these populations.

**Purpose:**

To examine how rurality relates to food insecurity and need for food resources, as well as availability of those resources including food pantries and soup kitchens, in 15 northern Ohio Appalachian counties.

**Methods:**

A cross-sectional study with a geographical analysis was conducted using data from the American Community Survey census data, County Health Rankings data, and regional foodbank websites.

**Results:**

Rural counties had a higher ratio of potential clients per service for food insecurity than did non-rural counties. They also had slightly more children eligible for free or reduced-price lunches than non-rural counties. However, the non-rural counties had slightly higher percentages of residents classified as food insecure and with limited access to healthy food.

**Implications:**

There are more potential clients per service for food insecurity in rural counties compared to non-rural counties. To promote greater access, additional food pantries should be opened in rural counties.

## INTRODUCTION

As of 2019, roughly 10.5% of all American households were food insecure, with 6.4% having low food security and 4.1% having very low food security.^1^ The USDA defines food insecure households as “uncertain of having or unable to acquire enough food to meet the needs of all their members because they have insufficient money or other resources for food.”^2^

Multiple factors are related to food insecurity including low income, limited access, and food costs. These issues tend to be greater in rural areas, with some research^3^ demonstrating that Appalachian areas had among the greatest food expense to income ratio in the U.S. Indeed, rural Appalachian areas experience food insecurity at even greater rates, with some estimates ranging from 23%^4^ to 29%^5^ (among individuals with household income of less than $20,000). Holben and others^6^ reported that among 808 participants from six Ohio Appalachian counties, food insecurity was three times higher than the rest of the Ohio population and food insecurity with hunger was seven times greater. Food insecurity among rural Appalachian populations is a critical issue, as it has been related to greater disease burden and chronic health conditions. For example, a cross-sectional survey of 1006 rural Appalachian respondents reports significantly poorer functional health among food insecure respondents compared to those who were not food insecure.^4^ Similarly, in Ohio, Appalachian individuals who were food insecure had significantly greater BMIs and rates of obesity.^6^

To combat this, households in Appalachian areas rely on government benefits, such as Supplemental Nutrition Assistance (SNAP), Special Supplemental Nutrition Program for Women, Infants and Children (WIC) and the National School Lunch Program (NSLP).^2^ Within Appalachian settings, rural and urban residents may differ in the ways they address food insecurity. For example, rural residents often rely on techniques such as not wasting food and food sharing networks.^7^ Nonmarket food exchanges tend to be more prevalent in rural populations. Rural populations also have greater access to gardens, which has a great impact on the consumption of fresh produce^8^ and rural Ohioans who garden tend to be less food insecure than their neighbors who do not.^9^ Additionally, rural Appalachian residents often rely on food pantries as a primary source of food while urban residents rely more heavily on programs such as SNAP.^8^

Food pantries and foodbanks are important components of the emergency food system.^10^ Food pantries and soup kitchens are defined as organizations that provide food directly to individuals and families, and foodbanks are defined as organizations that supply food stuff to food pantries, soup kitchens, and other organizations. Despite the primary role the emergency food system may play in addressing food insecurity in Appalachia, no studies have examined geographic differences in access to emergency food assistance in Appalachia. In this study, a geographic information system (GIS) approach was applied to examine how rurality relates to food insecurity and need for food resources, as well as availability of those resources including food pantries and soup kitchens, in 15 Northern Ohio Appalachian counties.

## METHODS

A cross-sectional study with a geographic analysis was conducted to examine 15 counties in Northern Ohio that are designated as part of Appalachia by the Appalachian Regional Commission.^11^ These counties include Ashtabula, Belmont, Carroll, Columbiana, Coshocton, Guernsey, Harrison, Holmes, Jefferson, Mahoning, Monroe, Muskingum, Noble, Trumbull, and Tuscarawas.

Services information (number of food pantries and soup kitchens) was obtained from the Ohio Association of Foodbanks partner websites in summer of 2019. Information from five foodbanks operating across the 15 counties were examined. Each foodbank website has services listed by county and lists were collected and the number of services were counted.

The following county aggregate information was also collected:

**County Total Population**. County total population was based on 2014–2019 data from the American Community Survey (ACS).^12^ The ACS is an on-going survey conducted by the U.S. Census to provide current information on demographic, economic, social, and housing topics.
**Number of Potential Clients Per Service**. Number of potential clients per service was calculated as the ratio of the total number of people living at or below 100% of the poverty line, based on 2014–2019 ACS data, per service within a county. The poverty line was used as a conservative estimate of the number of people at risk for food insecurity.
**Percent of Population Food Insecure**. The percent of the population of each county that is food insecure or does not have consistent access to food in the past year, was obtained from the 2017 Map the Meal Gap county estimates.^13^ The data are based on the U.S. Department of Agriculture (USDA) Food Security Survey 5-year estimates.
**Percent of Population with Limited Access to Healthy Food**. The percent of the population with limited access to healthy food was obtained from the 2015 USDA Food Environment Atlas.^14^ This statistic reports the percentage of the population with low income, defined as 200% or less of the federal poverty line, who live far from a grocery store. In rural settings, living within ten miles of a grocery store is defined as close to a grocery store, while living within one mile of a grocery store is categorized as close in urban settings.
**Percent of Children Eligible for Free or Reduced-Price Lunch**. The percent of public school children in preschool through 12th grade who are eligible for free or reduced-price lunch was obtained from the 2017–2018 County Health Rankings.^15^ Children are eligible for free lunch if their family income is 130% or less of the federal poverty level, and reduced lunch if the income is 180% or less of the federal income level.
**Rurality Level**. Counties were categorized by level of rurality based on Rural–Urban Continuum Codes.^16^ The following ten counties with a Rural–Urban Continuum Code greater than or equal to four were labeled as rural: Ashtabula, Columbiana, Coshocton, Guernsey, Harrison, Holmes, Monroe, Muskingum, Noble, and Tuscarawas. The remaining five counties were labeled as nonrural.

Using SPSS version 27,^17^ descriptive statistics were calculated, including the mean number of potential clients per service, the percent of food insecure, the percent with limited access to healthy food, and the percent of children eligible for free or reduced fee lunches. T-tests were used to compare mean differences between rural and nonrural counties.

ESRI ArcGIS Online was used to create maps for this analysis.^18^ County level data were spatially joined to Ohio county shape files from the U.S. Census Bureau using Federal Information Processing Standards (FIPS) codes. A choropleth map of RUCC codes was made to depict the rurality of the counties. Proportional symbols depicting the number of potential food bank clients were also added to the map. These data were spatially joined to the county shape files.

## RESULTS

Rural counties on average had a higher number of potential clients per service than did nonrural counties (1,097.30 versus 803.63 residents) as shown in Table 1. However, the difference was not statistically significant (p > 0.05). They also had a slightly higher percentage of children who were eligible for free or reduced-price lunches (53.60% versus 51.20%). However, the nonrural counties had a higher percentage of residents who were classified as food insecure (15.40% versus 14.70%) and with limited access to healthy food (7.20% versus 6.50%) compared to the rural counties. These small differences were not statistically significant (p >0.05).

**Figure 1:**
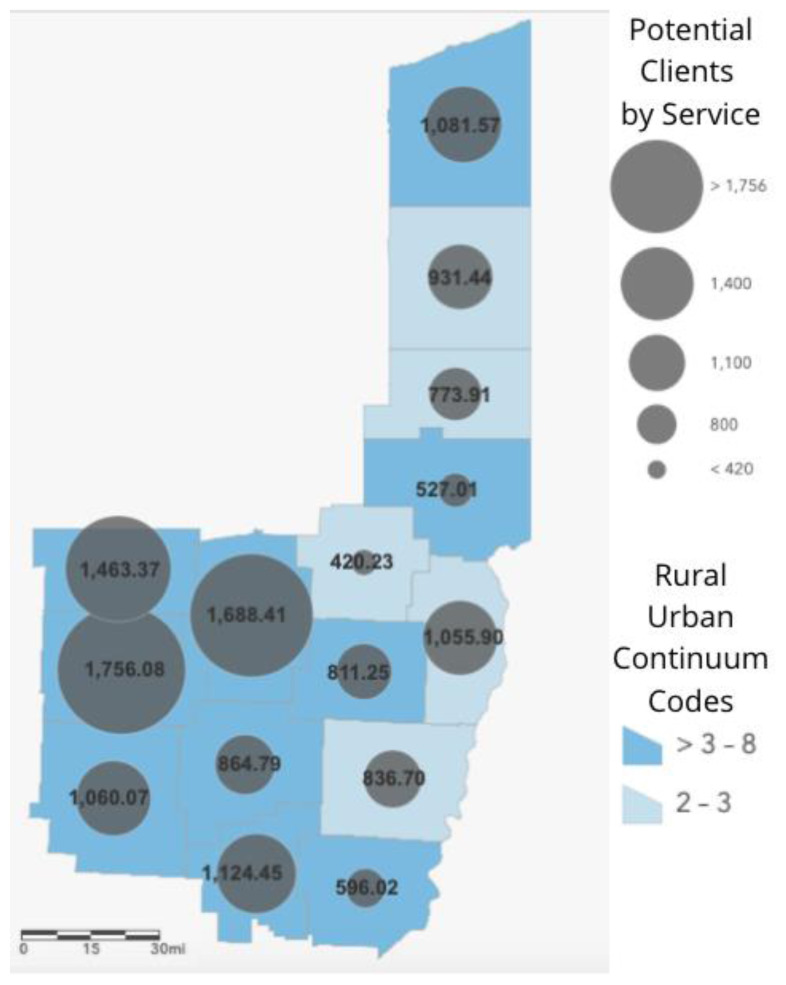
Rural–Urban Continuum Codes and the number of people living in poverty per service. The light blue counties are nonrural counties with Rural–Urban Continuum Codes (RUCC) from 1 to 3. The dark blue counties are rural counties, with RUUCs from 4 to 8. The circles on the map represent the number of people living in poverty compared to the number of services in each county; the larger the circle the higher the number of people each service must support. For example, each service in Mahoning County must work with 931 people whereas each service in Coshocton County must with 1830 people.

Figure 1 depicts the rurality of the counties as well as the number of services per population in poverty. The light blue counties are nonrural and have RUCC from 1 to 3. The dark blue counties are more rural with RUCC from 4 to 8. The grey circles represent the number potential clients per service in each county. The numbers also describe the number of potential clients per service in each county. For example, each service in Mahoning County must work with 575 potential clients whereas each service in Coshocton County must work with 1830 potential clients.

## IMPLICATIONS

This study found that, although the difference was not statistically significant, the rural counties had a higher number of potential clients per service than did nonrural counties. This is particularly troubling since some research shows that rural Appalachian residents often rely on food pantries as a primary source of food more so than their urban Appalachian residents who rely more heavily on programs such as SNAP.^4^ While it appears that there are similar levels of food insecurity across the region, some rural county residents may supplement their food by gardening and sharing food as suggested by Morton et al., which could lead to a decreased need for food pantries and other resources.^8^ Regardless, there is a large difference in geographic distribution of pantries and a lack of access to food pantries presents a problem for the food insecure. Additional work should be done to identify where these services are compared to the population in need. This study demonstrates the importance of combining geographic analysis with cross sectional analysis to identify gaps in services and to visually depict disparities.

## LIMITATIONS

There were several limitations to the current project. First, the lists of services may not be complete, and websites may be outdated.^19^ Additionally, because only 15 counties were examined, there was not enough power to find statistically significant differences between nonrural and rural counties. All findings should be interpreted cautiously. Further, current events, specifically the COVID-19 pandemic, has greatly increased food insecurity.^20^ Because the pandemic increased the need for these resources while also forcing many food pantries to close or limit operations, the foodbanks have utilized several methods to fill in the gaps, including relying on the National Guard and increasing funding.^13^ While the data in this analysis were collected before the start of the pandemic, it can help contextualize the framework in which newly food insecure individuals are living. Lastly, Holmes County has a high proportion of Amish residents, roughly 41%.^21^ This population may skew some of the county statistics. Compared to its neighbors, it has a significantly lower percent food insecure and percent of children eligible for free or reduced school lunches.

**Summary Box**

**What is already known about this topic?** Foodbanks are a vital resource for the 13% of Ohioans who are food insecure.

**What is added to this report?** This study compared the foodbanks in Northern Appalachian Ohio and the distribution of their partner food pantries in the counties they serve.

**What are the implications for future research?**

Future research is needed on the impact of the COVID-19 pandemic and related legislation on food insecurity.

## Figures and Tables

**Table 1:** Differences in rural and nonrural counties on food insecurity measures in the Northern Ohio Appalachian area (N = 15 counties)

	Nonrural (n=5 counties) Mean (SD)	Rural (n=10 counties) Mean (SD)	t-test	p value
Total Population	118,411 (90,419.28)	54,265 (36,734.90)	1.53	0.19
Percent of Population Living at or below the Poverty Line	15.36 (2.80)	15.60 (3.02)	−0.15	0.88
Number of Services	23.60 (19.83)	9.00 (8.46)	1.58	0.18
Potential Clients per Service	803.63 (239.24)	1097.30 (425.86)	−1.42	0.18
Percent of Population Food Insecure	15.40 (1.52)	14.70 (1.49)	0.85	0.41
Percent of Population with Limited Access to Healthy Food	7.20 (3.70)	6.50 (4.09)	0.32	0.75
Percent of Children Eligible for Free or Reduced-Price Lunch	51.20 (7.56)	53.60 (10.86)	−0.44	0.67
